# Reasonable Expectations of Privacy and Disclosure of Health Data

**DOI:** 10.1093/medlaw/fwaa004

**Published:** 2020-02-19

**Authors:** Mark J Taylor, James Wilson

**Affiliations:** f1 Associate Professor in Health Law and Regulation, Melbourne Law School, University of Melbourne, Australia; f2 Senior Lecturer, Department of Philosophy, UCL, London, UK

*Medical Law Review*, 2019, doi:10.1093/medlaw/fwz009

In the original version of this article, the authors wrote that:

*In one prominent case, Royal Free London NHS Foundation Trust shared the records of 1.6 million NHS patients with DeepMind for the development and testing of an App for detecting acute kidney injury, it argued, on the basis of implied consent for direct care. This was despite the fact that at the time that the data was shared, no steps were taken to make patients aware of this fact, and that only a small percentage of the 1.6 million patients would ever develop an acute kidney injury.*

The authors acknowledge that, given that the Royal Free London NHS Foundation Trust maintain that the sole purpose for sharing data with DeepMind was clinical testing, the below passage should replace the above original passage:

*In one prominent case, Royal Free London NHS Foundation Trust shared the records of 1.6 million NHS patients with DeepMind, for what it maintained to be clinical safety testing of an App for detecting acute kidney injury. It argued that it had done so on the basis of implied consent for direct care. This was despite the fact that at the time that the data was shared, no steps were taken to make patients aware of this fact, and that only a small percentage of the 1.6 million patients would ever develop an acute kidney injury.*

In addition, a new footnote 20 documenting the Information Commissioner’s use of the term ‘shared’ in relation to the patient records provided by the Royal Free London NHS Foundation Trust to Deepmind has been added after ‘Royal Free London NHS Foundation Trust shared the records of 1.6 million NHS patients with DeepMind’, as follows:

20 See the statement by the Information Commissioner quoted on the Information Commissioner’s Office website <https://ico.org.uk/about-the-ico/news-and-events/news-and-blogs/2017/07/royal-free-google-deepmind-trial-failed-to-comply-with-data-protection-law/> accessed on 7 January 2020.

All original footnotes remain unchanged, although the original footnote 20 becomes footnote 21 and so on.

The above changes have now been made to the paper.

